# Fuzzy-set qualitative comparative analysis of influencing factors on family doctor service performance during major public health emergencies

**DOI:** 10.3389/fpubh.2025.1565499

**Published:** 2025-04-08

**Authors:** Gan Wang, Li Luo

**Affiliations:** ^1^Shanghai Institute of Infectious Diseases and Biosecurity, Fudan University, Shanghai, China; ^2^School of Public Health, Fudan University, Shanghai, China

**Keywords:** family doctor, service performance, major public health emergencies, fsQCA, influencing factors

## Abstract

**Objective:**

By studying the Technology-Organization-Environment Framework (TOE), this research explores the impact of various indicators in technology, organization, and environment on the performance of family doctor services during major public health emergencies. It aims to identify the driving paths to improve performance.

**Methods:**

A stratified sampling of 34 community health service centers in Shanghai was conducted, using the comprehensive performance score of family doctors as the outcome variable. The Average Internet Medical Service Person-times and the Information Technology Expenditure per Thousand Population were considered as technology-related variables. The Fiscal Allocation per Thousand Population (/1,000), the Family Doctor Team Members per Thousand Population, and the Medical Social Workers and Volunteers per Thousand Population were identified as organization-related variables. The Proportion of Older Adult Population, Fiscal Allocation per Thousand Population, and the number of patient self-education organizations per thousand population were taken as environment-related variables. Fuzzy-set Qualitative Comparative Analysis (fsQCA) was employed to conduct necessity analysis, truth table analysis, and configurational analysis of antecedent conditions, with robustness tests performed by adjusting consistency thresholds and case frequencies.

**Results:**

The study found that the performance of family doctor services was influenced by multiple factors, with no single decisive factor. In overall communities, five configurations, including per capita fiscal allocation and community participation, affected performance, explaining 4.2% of the variance. In central urban areas, information technology expenditure and the Proportion of Older Adult Population were core conditions, influencing 27.5% of performance paths. In non-central urban areas, core conditions such as financial support and IT covered 53.9% of data cases. The fsQCA results, which were robustly tested, begin to provide a strong basis for resource allocation and policy formulation.

**Conclusion:**

This study begins to fill the gap in research on family doctor service performance during major public health emergencies, exploring the synergistic effects and causal asymmetry among multiple indicators such as technology, organization, and environment from a holistic, or configurational, perspective.

## 1 Introduction

Family doctors were on the frontline during the COVID-19 pandemic (1), managing not only their routine general practice services but also acute COVID-19 patients and those with long-term post-infection symptoms (2). For instance, Dutch general practitioners implemented various measures to reduce infection risks, such as minimizing personal contact, adjusting appointment systems and triage, and conducting as many remote consultations as possible (3). Despite playing a crucial role, family doctors worldwide faced challenges, including the complexity of coordinating management during the pandemic due to the diverse ownership structures of primary care facilities in the United States, ranging from state or government ownership to individual doctor ownership or community non-profit companies. Small independent clinics often struggled with shortages of personal protective equipment and a lack of hardware and software for virtual consultations, as well as decreased outpatient volume and service revenue (4). In England, the level of longitudinal continuity of care by family doctors dropped from 27.0 to 16.6% between 2018 and 2022 (5). The performance of family doctor services in emergency contexts was not only related to the continuity and efficiency of basic medical services but also directly affected the effectiveness of public health event prevention and control (6). The severity of these issues lay in their potential to weaken the “gatekeeper” role of family doctor teams in emergency prevention and control, thereby affecting the performance of the entire public health security system.

Exploring the factors affecting the performance of family doctors during the pandemic is crucial for improving the quality and efficiency of grassroots emergency medical services, optimizing the allocation of emergency medical resources, and enhancing the health levels of residents. Currently, there is a lack of research on the factors affecting the performance of family doctors during major public health emergencies. This study employs Fuzzy Set Qualitative Comparative Analysis (fsQCA) to examine the impact of technology, organization, and environment factors within the Technology-Organization-Environment (TOE) framework on the performance of family doctors during major public health emergencies, identifying the driving paths for improving family doctor service performance.

Specifically, this study aims to address three questions: What are the condition configurations that promote the improvement of family doctor service performance? Which conditions are more important for enhancing family doctor service performance? Are there differences in the performance of family doctors in urban and non-urban areas of Shanghai? Referring to the TOE framework, the study constructs a research framework for the factors affecting family doctor service performance and discusses the condition configurations and mechanisms causing regional differences in family doctor service performance based on the practical situations of family doctor service provision in 34 community health centers across 16 districts in Shanghai. This study helps to broaden the perspective of research on family doctor service performance and deepens the understanding of the driving paths and mechanisms of family doctor service performance, promoting the comprehensive realization of family doctor service performance during the pandemic.

## 2 Theoretical background and framework

The TOE framework, which encompasses technology, environment, and organization, is feasible for evaluating the performance of family doctors during major public health emergencies. It helps to identify and understand how technological, organizational, and environmental factors affect the response and service effectiveness of family doctors. Technology factors include relative advantage, compatibility, and complexity, such as the hardware facilities and internet skills invested by the institution. Organizational factors refer to the scope, scale, structure, and human resource conditions of the institution. Environmental factors include economic, cultural, and policy aspects (7). Studies have already used the TOE model to guide research in the medical field, such as constructing a framework for factors affecting the implementation of value-based medical performance assessment, involving key factors like medical information system construction, management support and commitment, staff-specific training, patient costs, and health insurance payment system reform (8).The TOE framework has also been used to explore the determinants of hospitals’ adoption of mobile healthcare, with perceived ease of use, system security, top management support, hospital size, and external pressure significantly related to the adoption of mobile healthcare by hospitals (9). Additionally, information technology infrastructure, system reliability, and government policies are significantly related to the adoption of mobile healthcare by hospitals, but with a negative correlation. There are also studies using the TOE framework to conduct a questionnaire survey on the potential factors affecting doctors’ willingness to use clinical practice guidelines for antimicrobial drugs (10). Therefore, the TOE framework, as a commonly used analytical framework, can be used to assess the factors affecting organizational performance. Here is how to use the TOE framework for evaluation.

### 2.1 Technology-related conditional variables

During the COVID-19 pandemic, telehealth services have become a key measure to reduce face-to-face contact and optimize the allocation of medical resources. Studies have shown that acceptance of telehealth varies across different population groups: younger individuals, non-immigrants, and women are more likely to choose telehealth, while the older adult prefer telephone consultations. Younger, wealthier, and more educated individuals are more inclined towards online medical consultations, and women use these services slightly more often than men. Although individuals from wealthier regions may be more likely to choose digital consultations, this trend remains inconclusive (11).

Against this backdrop, governments worldwide have adjusted policies to support telehealth services. For example, Australia has increased “bulk billing” for virtual medical services and clarified billing procedures to streamline funding for internet-based medical services (12). In the United States, the CARES Act allocated $8.7 million for telehealth technologies, focusing on rural and underserved areas to enhance telehealth capabilities (13). WHO’s Operational Framework for Primary Health Care highlights several “levers” to optimize services, with a particular focus on leveraging digital technology to enhance the effectiveness and efficiency of health services (14).

Based on this context, this study selects two technology-related indicators to measure technological conditions: “Average Internet Medical Service Person-times” and “Information Technology Expenditure per Thousand Population.” The prolonged pandemic has spurred a surge in demand for online medical services, shifting healthcare-seeking behavior from offline to online platforms (15). The indicator “Average Internet Medical Service Person-times” accurately reflects the frequency and coverage of internet-based medical services, capturing changes in patient behavior driven by technological applications and their potential impact on the trust relationship between patients and doctors (16). The indicator “Information Technology Expenditure per Thousand Population” reflects investment in information technology infrastructure, which is crucial for supporting telehealth services and digital health management. These two indicators provide an important basis for evaluating the impact of technological conditions on the performance of family doctor services.

### 2.2 Organization-related conditional variables

Incorporating economic principles into decision-making is crucial for preparing countries to address the significant and unexpected supply and demand shocks characteristic of pandemics. During the COVID-19 pandemic, the Australian government swiftly implemented fiscal stimulus measures to support the labor market (17). The Reserve Bank of Australia also launched significant monetary policy actions to improve capital market liquidity, reducing the cash rate target to a historical low to facilitate nationwide lending and increase support for individuals and businesses (17). Additionally, the Australian government invested $2.4 billion to establish more respiratory clinics and $10 million for COVID-19 nucleic acid testing (18). These measures highlight the importance of fiscal and organizational preparedness in mitigating the impacts of public health crises.

In England, as of November 2024, there were 38,508 fully qualified general practitioners (GPs) working in the NHS, equivalent to 28,139 full-time GPs. Since 2015, the total number of GPs has seen minimal growth, while the number of GP partners has significantly declined (19). In 2019, the government committed to adding 6,000 GPs by 2024, but only 3,697 have been added since 2019. The number of fully qualified full-time GPs decreased by 1,226 since 2015, though it increased by 655 in the past year. The GP partner workforce has lost 5,927 full-time partners since 2015, with 529 lost in 2023 alone. Without appropriate government measures, these losses are expected to continue as general practice pressures mount (19). An adequate number of family doctors is essential for effective team collaboration and service delivery, making it a key organizational condition for family doctor services.

In China, the COVID-19 pandemic highlighted the importance of community engagement and volunteer participation in public health responses. After the outbreak, numerous volunteers were recruited through mobile apps to carry out urgent tasks such as transporting supplies and supporting frontline medical staff, assisting in official pandemic response efforts (20). The Chinese government increasingly recognizes the value of civil society organizations in providing social services and enhancing government legitimacy (21, 22). Cooperation between citizens, civil society, and local governments is crucial for filling the gaps in public services during health emergencies. Experienced local volunteers played a key role by swiftly shifting their focus to pandemic response efforts, demonstrating the importance of community involvement in mitigating public health crises (20).

Considering the differences in population size across jurisdictions and adhering to the principle of data standardization, this study selects three organizational conditional variables: “Fiscal Allocation per Thousand Population (/1,000),” “Family Doctor Team Members per Thousand Population,” and “Medical Social Workers and Volunteers per Thousand Population.” These variables reflect the financial support available for primary care, the capacity of family doctor teams, and the involvement of social workers and volunteers in healthcare delivery. They provide a comprehensive basis for evaluating the organizational conditions influencing family doctor service performance.

### 2.3 Environment-related conditional variables

The high prevalence of chronic diseases globally is largely driven by the aging population, a problem further highlighted during the COVID-19 pandemic (23). The pandemic not only increased the demand on healthcare systems but also limited their capacity to support and respond to the large number of older adult individuals, many of whom suffer from multiple chronic conditions. Strengthening primary care remains an effective strategy to reduce the incidence and mortality rates of chronic diseases and to control national healthcare expenditures (24, 25). International evidence shows that health systems focusing on Primary Health Care (PHC) achieve better health outcomes and lower medical costs (25). As the first point of contact with the healthcare system, PHC provides a comprehensive range of treatment services, as well as health promotion, prevention, and screening services that typically do not require referrals. During the COVID-19 pandemic, primary care played a significant role through prevention, timely diagnosis, referral of severe cases, and telehealth services.

The Person-Centered Primary Care Measure (PCPCM) has been validated across multiple cultural contexts, including in 35 OECD countries, and is widely used to evaluate the quality of primary care services (26). Among its components, community context is recognized as one of the core elements that patients value in community-based services (27). Community health service centers in Shanghai have implemented a new self-management model for non-communicable diseases (NCDs). Previous studies have shown that the implementation rate (80.79%) and effectiveness (86.66%) of chronic disease self-management among contracted patients were higher than those among non-contracted patients (55.57 and 54.79%, respectively) (28). Contracted patients were 2.25 times more likely to engage in self-management and 2.91 times more likely to report it as effective. Understanding family doctor contract services, satisfaction with community health centers, and positive initial contact experiences significantly influenced the implementation and effectiveness of self-management. Self-education can enhance patients’ understanding of disease management, thereby positively affecting the performance of family doctor services in emergency situations (see Table A1 in Appendix A).

Based on the above background, this study selects three environment-related conditional variables to comprehensively evaluate the performance of family doctor services. First, the “Proportion of Older Adult Population” is used to measure the coverage and demand satisfaction of family doctor services among the older adult group. Second, “Average Medical Expenses per Capita (/1,000)” reflects the role of family doctors in controlling medical costs and provides an important basis for evaluating the economic effectiveness of their services and the implementation of performance incentive policies. Finally, the “number of patient self-education organizations per thousand population” is used to measure the prevalence of patient health management. Self-education can enhance patients’ understanding of disease management, thereby positively affecting the performance of family doctor services in emergency situations. These indicators collectively reflect several key factors at the environmental level and provide an important basis for the comprehensive evaluation of family doctor service performance.

### 2.4 Outcome variable

**Figure 1:**
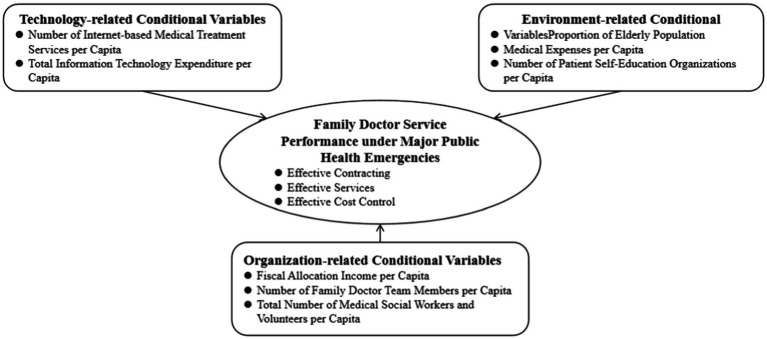
Family doctor service performance model in emergency context.

Shanghai has issued the “Key Performance Assessment Indicators for Family Doctor Contract Services in Shanghai (2022 Edition)” (29) to motivate and constrain family doctors through assessments. Based on the data from the Shanghai Health and Health Statistics Center, this study selected a total of 16 indicators for effective contracting, effective services, and effective cost control. Using the entropy method to determine the weight of each indicator, the comprehensive performance scores of family doctors in community health service centers are calculated and used as the result conditions for fsQCA analysis (Table B1 for Appendix B). Key insights on factors shaping family doctor service performance are detailed in Figure 1.

## 3 Methods

### 3.1 Data collection

In accordance with the “Shanghai Health Resources and Medical Service Statistical Survey System” (hereinafter referred to as the “Statistical Survey System”), researchers gathered monthly indicators of family doctor service provision from January 2020 to December 2023. Employing stratified sampling methods, 2 community health service centers were randomly selected from each of the 15 districts in Shanghai, excluding Pudong New Area. As of the end of 2023, the permanent resident population of Shanghai was 24.87 million, with Pudong New Area accounting for 23.36% (30). It is worth noting that, apart from Pudong New Area, the population differences among the remaining districts were relatively small (31). Given its larger population share, 4 community health service centers were randomly selected from Pudong New Area. In total, 34 community health service centers were included in the study. The data collection focused on four key sub-reports within the “Statistical Survey System”: Form 1 of the Annual Report on Health Institutions (Community Health Service Centers and Stations), Form 2 of the same report, the Home Care Bed Work Situation report, and the Monthly Report of Medical Institutions.

During the COVID-19 outbreak in Shanghai, a city-wide lockdown was implemented from March 2022 to May 2022 (32). During this period, certain monthly indicators might have been affected by the lockdown measures. However, the indicators ultimately included in the fsQCA calculations were all annual averages. Moreover, the lockdown and subsequent lifting of restrictions in Shanghai’s districts were relatively synchronized. Therefore, the impact of the lockdown on the values of these indicators was minimal. For districts where monthly indicator values were missing during the lockdown period, this study used monthly averages to impute the missing values.

### 3.2 Fuzzy set qualitative comparative analysis

This study employed fsQCA to investigate the comprehensive impact of the TOE framework elements on the performance of family doctors during major public health emergencies. fsQCA was an ideal tool for this type of analysis as it revealed how different configurations of conditions influenced specific outcomes and identified key combinations of conditions that led to these outcomes (33). The fsQCA process included calculating membership scores, identifying core and peripheral conditions, constructing logical models, analyzing configurations, revealing interactive effects and driving paths, and discussing and validating the findings. It emphasized that not all conditions were necessary to explain outcomes, but specific combinations of conditions could be sufficient (34).

A quality control process for the use of fsQCA was established in this study to demonstrate its applicability (Table C1 for Appendix C). Additionally, the study compared the overall sample of Shanghai with samples from the central and non-central urban areas. The central urban area of Shanghai, covering approximately 664 square kilometers within the outer ring road, includes districts such as Huangpu, Xuhui, Changning, Jing’an, Putuo, Hongkou, Yangpu, and parts of Pudong New Area, Minhang, Baoshan, and Jiading. The central city area is defined by the overall urban planning regulations, with areas outside the central city considered suburban (35). Economic development, geographical location, and resource endowment, especially internet-based medical services, total information expenses, per capita fiscal allocations, and the Proportion of Older Adult Population, significantly influence the performance of family doctors.

**Figure 2:**
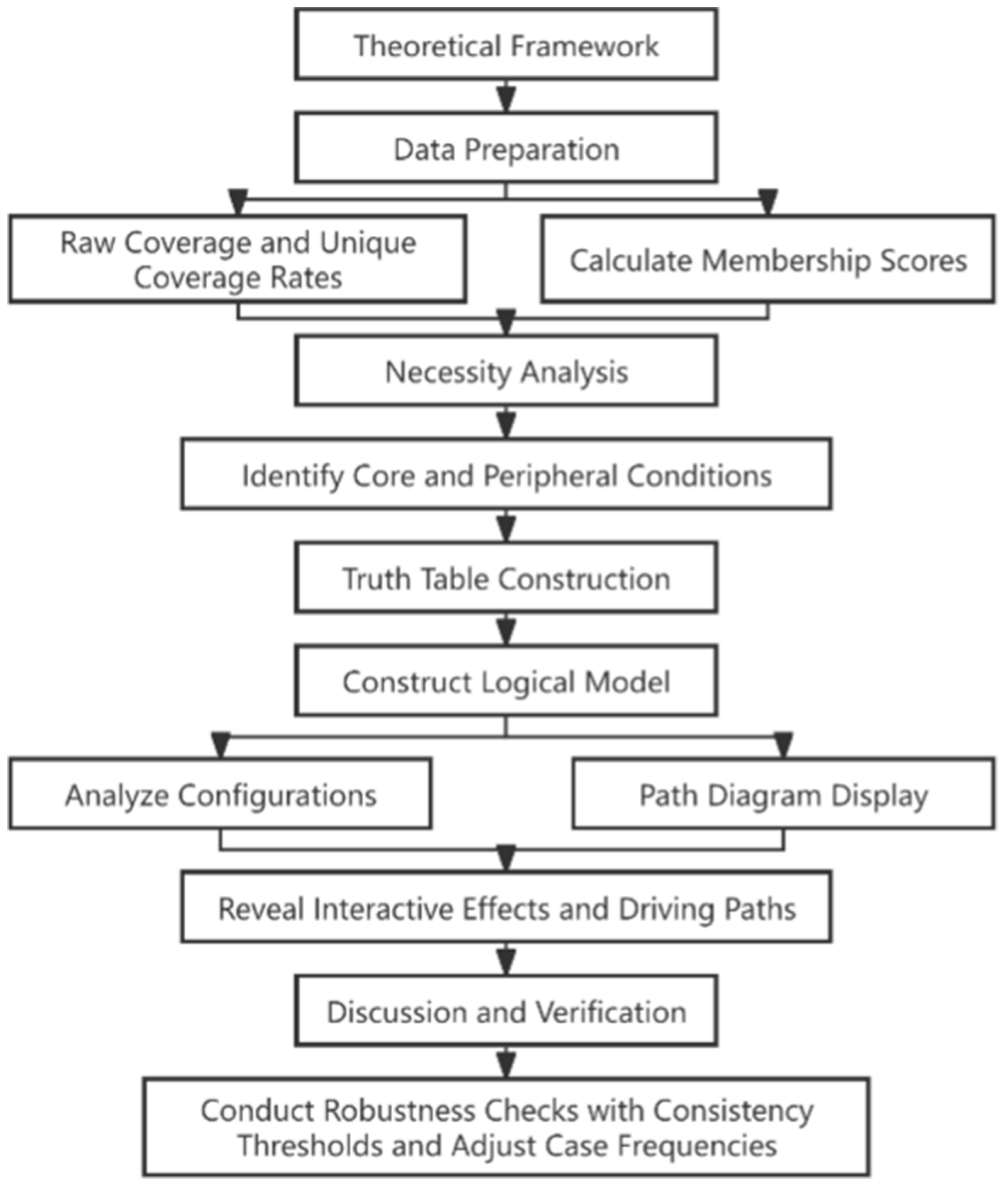
Analysis path of influencing factors on family doctor service performance in emergency context using fsQCA.

This study standardized all indicators based on the population of the jurisdictions where each institution was located. To ensure the reliability and scientific validity of the study and to mitigate the impact of random events on the research findings, this study conducted robustness tests by altering the consistency threshold and adjusting the frequency of case numbers (36) (Figure 2).

### 3.3 Calibration of set membership

In fsQCA, each condition and outcome is considered as an independent set, and each case has membership scores in these sets. After standardizing the raw data to eliminate the impact of different scales and magnitudes on the evaluation results, the process of assigning set membership scores to cases is known as calibration. This paper drew on existing research, based on existing theories and empirical knowledge, and according to the data types of each condition and outcome, used the direct calibration method to transform data into fuzzy set membership scores (36). In direct calibration, the determination of thresholds facilitates the reproducibility and validation of studies and enables direct comparison and further analysis with other results found in the literature. According to the commonly used percentile method, the calibration threshold was set at the 0.5 percentile, the non-membership calibration threshold at the 0.05 percentile, and the full membership calibration threshold at the 0.95 percentile. Additionally, the calibration standard for the attention allocation crossover point was typically set at 0.5, the full membership calibration standard at 0.05, and the non-membership calibration standard at 0.95 (37) (Table 1).

**Table 1:** Calibration of conditions and outcomes.

Conditions/Outcomes		Calibration		
		Full membership	Cross-point	Non-membership
Outcome Variable	Performance of Family Doctor Services	0.061	0.001	0.0001
Technical Conditions	Average Internet Medical Service Person-times	80.900	23.000	2.300
	Information Technology Expenditure per Thousand Population (/1,000)	0.985	0.355	0.130
Organizational Conditions	Fiscal Allocation per Thousand Population (/1,000)	1.661	0.555	0.183
	Family Doctor Team Members per Thousand Population	3.301	0.905	0.140
	Medical Social Workers and Volunteers per Thousand Population	0.410	0.267	0.128
Environmental Conditions	Proportion of Older Adult Population	1.939	1.120	0.210
	Average Medical Expenses per Capita (/1,000)	0.361	0.115	0.010
	Number of Patient Self-Education Organizations per Thousand Population	0.300	0.190	0.036

### 3.4 Truth table preparation

The frequency of a configuration indicates how many cases in the sample can be explained by that specific combination of conditions. If the frequency is zero, it means no cases in the sample can be explained by that combination. To ensure meaningful and valid results, this study set a frequency threshold of 1 for the truth table analysis of family doctor service performance in overall, central urban, and non-central urban communities, and removed all combinations with lower frequencies (38, 39). After filtering out uncommon configurations, the truth table was sorted by raw consistency and PRI consistency to identify combinations that score high on the outcome variable.

This study set a critical value of 0.8 for raw consistency and 0.7 for PRI consistency, meaning only combinations with a raw consistency of at least 0.8 and a PRI consistency of at least 0.7 are considered reliable in leading to perceived accountability (37, 40). When a configuration’s raw consistency is above 0.8 and its PRI consistency is above 0.7, the outcome variable is assigned a value of 1, indicating the combination can explain the outcome; otherwise, it is assigned a value of 0, indicating it cannot explain the outcome. To identify the core and complementary conditions influencing family doctor service performance during significant public health events, both parsimonious and intermediate solutions were considered. This research compiled causal recipes for family doctors across all community health centers and illustrated the recipes for achieving high service performance in both high- and low-complexity hospitals.

Consistency and coverage scores are key metrics for validating solutions within fsQCA. Coverage scores indicate the proportion of the outcome’s members explained by each solution, while overall coverage indicates how well the outcome can be accounted for by the configurations. Higher coverage scores suggest empirical relevance and effectiveness, though they do not necessarily denote theoretical significance (33). Moreover, this study conducted robustness testing by altering the consistency threshold and adjusting case frequencies (36, 41).

## 4 Results

### 4.1 Necessity analysis

Upon analyzing the necessary conditions for family doctor service performance across overall, central urban, and non-central urban communities, it was found that the consistency, serving as a benchmark for necessary conditions, was below 0.9 in each instance. Consequently, there were no essential conditions that significantly influenced either high or non-high family doctor performance. This suggested that the achievement of community family doctor service performance was not dictated by a single factor but was rather an outcome of the interplay of multiple factors (Table D1-3 for Appendix D).

### 4.2 Truth table analysis

#### 4.2.1 Configurational analysis of antecedent conditions for overall community family doctor service performance

The study’s five configurations all exceeded the minimum acceptable consistency level of 0.75, with an overall solution consistency of 0.868 and coverage of 0.745. Configuration 1, with core conditions like per capita fiscal allocation and community participation, significantly influenced medical service quality and efficiency. It covered 24.9% of performance paths with a 0.993 consistency, explaining 4.2% of the variance. Core conditions across configurations highlighted the importance of informational investment, team size, community involvement, fiscal support, and the proportion of older adult people. The analysis revealed synergistic effects among technical, organizational, and environmental factors, such as the combination of high IT expenditure and team size enhancing service performance. It also showed the threshold effect for certain conditions like fiscal allocation, beyond which performance improved significantly. This fsQCA analysis provided insights into the complex interactions between factors, aiding in resource allocation and policy formulation for enhancing family doctor service performance (Table 2).

**Table 2:** Configurational analysis of antecedent conditions for overall community family doctor service performance.

Condition Variables	Technology-Organization-Environment Type									Technology-Organization Type	Technology-Environment Type			Organization-Environment Type		Organization Type
	Config 1	Config 2	Config 3	Config 4	Config 5	Config 6	Config 12	Config 15	Config 16	Config 10	Config 8	Config 9	Config 11	Config 13	Config 14	Config 7
Average Internet Medical Service Person-times	●	◯	○	●	●	●	○	●	◯	●	●	○	○	◯	◯	◯
Information Technology Expenditure per Capita (/1,000)	●	●	⬤	⬤	⬤		●	●	⬤	○	○	⬤	○	○	○	○
Fiscal Allocation per Capita (/1,000)	⬤	⬤		●	●	●	○	○	⬤	○	○	○	○	⬤	⬤	⬤
Family Doctor Team Members per Thousand Population	◯	●	●	●	●	●	○	●	●	●	○	○	○	◯	●	◯
Medical Social Workers and Volunteers per Thousand Population	⬤	⬤	⬤	⬤		⬤	⬤	○	○	⬤	○	○	○	⬤	⬤	○
Proportion of Older Adult Population	⬤	⬤	⬤	⬤	⬤	⬤	⬤	⬤	○	○	⬤	○	⬤	⬤	○	○
Average Medical Expenses per Capita (/1,000)	●	●	●		●	●	●	●	●	○	○	○	●	●	○	○
Number of Patient Self-Education Organizations per Thousand Population			⬤	⬤	⬤	●	○	○	⬤	○	○	⬤	●	○	●	○
Raw Coverage	0.249	0.326	0.293	0.205	0.229	0.268	0.210	0.185	0.247	0.178	0.181	0.193	0.193	0.214	0.174	0.212
Unique Coverage	0.042	0.034	0.030	0.007	0.014	0.059	0.017	0.020	0.014	0.008	0.017	0.003	0.016	0.016	0.000	0.000
Consistency	0.993	0.964	0.955	0.992	0.992	0.989	0.997	0.997	0.955	0.813	0.926	0.928	0.994	0.987	0.899	0.813
Overall Coverage	0.745															
Overall Consistency	0.868															

1. “Config” is the abbreviation for “Configuration”; 2. ⬤ or ● indicates the presence of a condition, ◯ or ○ indicates the absence of a condition; ⬤ or ◯ indicates a core condition, ● or ○ indicates a peripheral condition. Blank spaces represent conditions that may or may not exist.

#### 4.2.2 Configurational analysis of antecedent conditions for central urban community family doctor service performance

The study’s three configurations all exceeded the minimum consistency threshold of 0.75, with an overall solution consistency of 0.962 and coverage of 0.758. Configuration 1, with an original coverage of 0.275, explained 27.5% of the family doctor service performance paths, with a consistency of 0.994 and unique coverage of 0.061, indicating it explained 6.1% of the performance with high consistency and strong data coverage. Core conditions identified included information technology expenditure as a key factor in all configurations, highlighting the importance of IT investment in enhancing service performance in central urban areas. The Proportion of Older Adult Population was also a core condition in all configurations, directly affecting service performance. The combination of high IT expenditure and a high proportion of older adult people suggested a significant impact on service performance, while the marginal status of family doctor team numbers may have indicated competition with other conditions under limited resources. The analysis revealed the complex interactions between factors, providing a basis for optimizing resource allocation and policy formulation to enhance service performance (Table 3).

**Table 3:** Configurational analysis of antecedent conditions for family doctor service performance in central urban communities.

Condition variables	Technology-organization-environment type								Technology-organization type			Organization-environment type
	Config 1a	Config 2a	Config 3a	Config 4a	Config 8a	Config 10a	Config 11a	Config 12a	Config 5a	Config 6a	Config 7a	Config 9a
Average Internet Medical Service Person-times	●	●	●	●	◯	●	◯	◯	●	◯	◯	◯
Information Technology Expenditure per Capita (/1,000)	⬤	⬤	⬤		⬤	⬤	⬤	⬤	○	⬤	○	○
Fiscal Allocation per Capita (/1,000)	●	●	●	●	○	○	●	○	○	○	○	●
Family Doctor Team Members per Thousand Population	○	●	●	●	○	●	●	●	○	○	○	○
Medical Social Workers and Volunteers per Thousand Population	●	●		●	●	○	○	●	○	○	○	●
Proportion of Older Adult Population	⬤	⬤	⬤	⬤	⬤	⬤	○	⬤	⬤	○	⬤	⬤
Average Medical Expenses per Capita (/1,000)	●		●	●	●	●	●	●	○	○	●	●
Number of Patient Self-Education Organizations per Thousand Population		⬤	⬤	⬤	○	○	⬤	⬤	○	⬤	⬤	○
Raw Coverage	0.275	0.233	0.269	0.306	0.210	0.178	0.232	0.245	0.162	0.191	0.209	0.211
Unique Coverage	0.061	0.010	0.020	0.078	0.024	0.029	0.022	0.043	0.029	0.005	0.024	0.040
Consistency	0.994	0.996	0.994	1.000	1.000	1.000	0.993	0.990	0.937	0.923	0.996	1.000
Overall Coverage	0.758											
Overall Consistency	0.962											

1. “Config” is the abbreviation for “Configuration”; 2. ⬤ or ● indicates the presence of a condition, ◯ or ○ indicates the absence of a condition; ⬤ or ◯ indicates a core condition, ● or ○ indicates a peripheral condition. Blank spaces represent conditions that may or may not exist.

#### 4.2.3 Configurational analysis of antecedent conditions for non-central urban community family doctor service performance

One type of configuration presented exceeded the minimum consistency threshold of 0.75, with an overall solution consistency of 0.933 and coverage of 0.539. Core conditions included IT expenditure, per capita fiscal allocation, family doctor team size, community synergy, older adult population ratio, and per capita medical expenses, emphasizing the importance of financial support, IT, primary healthcare, community involvement, and older adult health needs. Marginal conditions like internet medical service person-times and patient self-education organization numbers also contributed to service accessibility and health awareness. The configuration achieved a unique coverage rate of 53.9%, covering 53.9% of the data cases. This indicates its significant and distinctive contribution to explaining the data. Specifically, this coverage rate suggests that over half of the high-performance cases in family doctor service supply in non-central urban areas can be explained by a single solution. High consistency near 1 suggested strong reliability in explaining outcomes. This analysis revealed the synergistic effects of technical, organizational, and environmental factors on service performance and provided a basis for optimizing resource allocation and policy formulation (Table 4).

**Table 4:** Configurational analysis of antecedent conditions for high family doctor service performance in non-central urban areas.

Condition variables	Technology-organization-environment type
	Config 1b
Average Internet Medical Service Person-times	○
Information Technology Expenditure per Capita (/1,000)	⬤
Fiscal Allocation per Capita (/1,000)	⬤
Family Doctor Team Members per Thousand Population	⬤
Medical Social Workers and Volunteers per Thousand Population	⬤
Proportion of Older Adult Population	⬤
Average Medical Expenses per Capita (/1,000)	⬤
Number of Patient Self-Education Organizations per Thousand Population	●
Raw Coverage	0.539
Unique Coverage	0.539
Consistency	0.933
Overall Coverage	0.539
Overall Consistency	0.933

1. “Config” is the abbreviation for “Configuration”; 2. ⬤ or ● indicates the presence of a condition, ◯ or ○ indicates the absence of a condition; ⬤ or ◯ indicates a core condition, ● or ○ indicates a peripheral condition. Blank spaces represent conditions that may or may not exist.

### 4.3 Rigor and robustness testing of fsQCA results

The results indicated that when the consistency threshold was modified from 0.8 to 0.85, 0.9, and 0.95, respectively, there were no differences in the robustness test outcomes of the initial configuration analysis. Researchers adjusted the frequency threshold from 1 to 2 to ensure that each configuration included a sufficient number of cases. These adjustments did not lead to significant changes in the number or composition of the configurations, indicating that our results are robust and reliable under varying parameter settings.

In this study, certain indicators such as Information Technology Expenditure per Capita (/1,000), Fiscal Allocation per Capita (/1,000), and Number of Patient Self-Education Organizations per Thousand Population frequently appeared as core conditions. This highlights their critical role in shaping the performance of family doctor services during the COVID-19 pandemic. This finding is consistent with previous literature, which has identified information technology funding, medical fiscal allocation, and the number of patient self-education organizations as important factors influencing the performance of family doctor services (14, 18, 28). The results further validated these theoretical propositions. The connection between empirical findings and theoretical justifications not only underscores the robustness of our analysis but also reinforces the importance of considering these elements in health service policy-making.

The study revealed that the performance of family doctor services is influenced by multiple factors, with no single decisive factor. In central urban areas, core conditions such as information technology expenditure per capita and the Proportion of Older Adult Population influenced 27.5% of the performance pathways. In non-central urban areas, core conditions like financial support and information technology expenditure per capita accounted for 53.9% of the data cases.

## 5 Discussion

Information and communication technology (ICT) is increasingly being utilized to enhance the accessibility of medical information and services, thereby improving self-management of diseases. The volume of health information transmitted through digital media is growing (42). For instance, digital health literacy, a concept reflecting changes and demands in the healthcare environment, refers to an individual’s knowledge and skills to use digital technology and health information to improve health (43). A study indicated that 42.0% of older adult cancer patients’ digital health literacy is significantly influenced by digital informatization (ss = 0.59, *p* < 0.001) and self-efficacy (ss = 0.20, *p* = 0.003), suggesting that strategies emphasizing digital health literacy, level of informatization, and self-efficacy can improve digital health literacy among older adult cancer patients (44).

National health informatization projects are extremely complex and costly, with the Chinese government playing a crucial supportive role: local medical service prices have not increased, and data privacy and usability have been better ensured (45). In 2022, the proportion of hospitals with investment in informatization construction between 2 million and 5 million RMB (inclusive) was the highest, reaching 20.53%, followed by those with annual investments between 5 million and 10 million RMB (inclusive), accounting for 15.16%. Among the surveyed hospitals, the average investment in informatization construction from 2021 to 2022 was 9.36 million RMB, an increase of 1.61 million RMB compared to the average of 7.75 million RMB from 2019 to 2020 (46). In 2020, the country successively introduced a series of policies to strengthen the supporting role of informatization in the COVID-19 pandemic, requiring the enhancement of data collection, analysis, and application, active development of remote medical services, standardization of internet diagnosis and consultation services, deepening “internet+” government services, and strengthening infrastructure and security protection (47). Therefore, investment in informatization is crucial for improving medical service supply and efficiency.

Volunteer services have the nature of “voluntariness” (48). Volunteers desire recognition and respect from those around them; thus, focusing on emotional factors in social mobilization can mobilize more potential social forces (49). The motivation of Chinese emergency volunteers stems more from emotional bonds and shared values, serving to feedback to the public (50). The country issued the “Guidelines for Volunteer Organizations and Volunteers to Participate in Epidemic Prevention and Control” during the pandemic, encouraging volunteer organizations and volunteers to participate orderly in services while protecting themselves, especially emphasizing “caring for frontline medical staff, community workers, families of the deceased, people with special difficulties, and the floating population, “and requiring assistance in medical treatment, community prevention and control, material distribution, psychological aid, and companionship and care for special key groups (51). At the same time, the Ministry of Civil Affairs’ “Notice on Further Mobilizing Professional Social Work Forces to Participate in Epidemic Prevention and Control Work” called for “promoting the linkage mechanism between communities and social organizations, social workers, community volunteers, and social charity resources, “effectively combating the COVID-19 pandemic (52).

Shanghai has provided appropriate temporary work allowances to community workers involved in community COVID-19 prevention and control efforts, and has offered temporary subsidies to community volunteers (53). In addition, China Life Insurance Company Limited, Shanghai Branch, has provided a “Guardian Volunteer” special insurance for volunteers participating in COVID-19 prevention and control service projects on the “Shanghai Volunteer Network.” On top of the existing coverage, this insurance offered a maximum of RMB 200,000 per person for accidental death during volunteer service (including travel to and from the service site) and an additional RMB 500,000 per person for death caused by confirmed COVID-19 infection. Volunteers were also awarded a “Shanghai COVID-19 Prevention and Control Volunteer Service Certificate.” (54) In a gesture of profound respect and heartfelt gratitude, the Shanghai Municipal Government has granted a one-time, phased allowance of RMB 6,000 to each frontline medical worker, in recognition of their exceptional contributions to the epidemic response efforts (55).

Shanghai, being a super-aged society, has 5.68 million people aged 60 and above out of a total household population of 15.1947 million, accounting for 37.4% of the total population; 4.3792 million people aged 65 and above, accounting for 28.8% of the total population; and 0.8164 million people aged 80 and above, accounting for 5.4% of the total population (56). By the end of 2023, there were 1.783 million older adult people in “pure older adult households” in the city, including 0.331 million people aged 80 and above in “pure older adult households”; the number of solitary older adult people was 0.3341 million, including 0.0258 million older adult people without family (56). During major public health emergencies, Shanghai, as a super-aged society, has a high proportion of older adult population, which significantly increases the demand for family doctor services. The abundance of community resources, such as day-care centers and meal service points, provides strong support for family doctors. In addition, the collaborative efforts of professional personnel and volunteers, as well as the protection of long-term care insurance, help enhance the service capacity and performance of family doctors in responding to public health emergencies.

Self-management support is not just about providing information to patients. It also involves committing to patient-centered care, offering comprehensive patient education, creating clinical teams composed of clinicians and administrators with clearly defined roles and responsibilities, and using office systems to support follow-up and tracking of patients. Self-management support is considered the least implemented and most challenging area to integrate into routine care in chronic disease management (57). It includes providing compassionate, patient-centered care; involving the entire care team in planning, executing, and following up on patient visits; focusing on prevention and care management rather than acute care during patient visits; engaging patients in goal setting; offering customized education and skill training using materials suitable for different cultures and health literacy levels; recommending community resources, such as programs to help patients quit smoking or follow exercise plans; and regularly tracking patient contact through emails, phone calls, text messages, and mail to support their efforts to maintain healthy behaviors (58). Self-management support is crucial, as patient self-management during pandemics helps reduce hospitalization rates (59).

The paper abandoned the traditional statistical method based on the “independent variable--dependent variable” binary relationship, instead adopting the fsQCA method. To reveal the pathways for enhancing family doctor service performance during major public health emergencies, it is insufficient to rely solely on analyzing single factors such as technology, organization, and environment or their simple interactions. Compared to traditional analysis techniques, fsQCA emphasizes the interdependence of causal conditions and the multiple conjunctural causations formed by different combinations, which helps to gain a deeper understanding of the drivers behind the differences in service performance of family doctors across districts in Shanghai during emergencies (40). Therefore, the fsQCA method is more suitable for exploring how multiple factors collectively influence family doctor service performance in emergency states from a holistic relationship perspective.

## 6 Limitations and future research

While this study selected some indicators related to technology, organization, and environment, it is uncertain whether these indicators comprehensively cover all key factors affecting the performance of family doctor services. In terms of technology, it could also consider the application and impact of emerging technologies like artificial intelligence and big data on performance. Regarding organization, further exploration is needed on how internal team structure, management mechanisms, and incentive policies affect performance. And in terms of environment, more factors such as policy, culture, and social support networks should be considered for their influence on performance. In fsQCA research, the selection of outcome variables and antecedent conditions is crucial. To enhance the precision of this selection process, Structural Equation Modeling (SEM) can be employed prior to fsQCA. SEM is capable of handling multiple outcome variables and latent variables simultaneously, making it suitable for analyzing complex relationships among variables. Through confirmatory factor analysis and path analysis within SEM, key variables that significantly influence the outcome variable and their interactions can be identified (60).

In the fsQCA results of this study, an important configurational pathway was identified: per capita expenditure on information technology and the number of patient self-education organizations per thousand population. When these two factors coexist, they generate a significant synergistic effect. On one hand, information technology provides patient self-education organizations with efficient channels for dissemination, such as online platforms and social media for health education. On the other hand, patient self-education organizations enhance residents’ health literacy, which in turn increases their acceptance and utilization of information technology tools (61, 62). This synergy enables family doctors to more effectively leverage information technology to provide continuous, high-quality medical services to community residents, especially during public health emergencies when routine medical services are restricted. Similarly, the significance of investing in telemedicine and other information technologies has been demonstrated in the UK and has received long-term government funding. For example, telecommunication companies such as BT, Virgin Media, and Sky have developed telemedicine services for the National Health Service (NHS), covering areas including primary care, clinical trials, consultations, and chronic disease assessments (61–63).

Although fsQCA is suitable for analyzing complex causal relationships, its results rely on researchers’ subjective judgments, such as the adjustment of consistency thresholds and case frequencies, which may lead to different conclusions among researchers. Future research could combine fsQCA with other methods like system dynamics and network analysis to more comprehensively examine the factors influencing family doctor service performance and their dynamic processes.

While the study offers some targeted suggestions for differences between regions (such as central and non-central urban areas), it does not delve into the deeper reasons behind these differences, such as the roles of policies and culture. The 16 driving paths and 5 adaptation models identified, though theoretically valuable, may face many challenges in practical application. For instance, how to balance the relationships between different conditions and how to prioritize and implement certain conditions under limited resources are issues not fully discussed in the abstract. Future research should focus on exploring strategies and paths to specifically implement these factors to enhance performance.

## 7 Conclusion

This study begins to fill the gap in research on family doctor service performance during major public health emergencies by exploring the synergistic effects and causal asymmetry among multiple indicators—such as technology, organization, and environment—from a holistic, configurational perspective.

Considering the resource constraints during major public health emergencies, this study suggests that the configuration combining Information Technology Expenditure per Capita and the Number of Patient Self-Education Organizations per Thousand Population (a technology-environment configuration) is relatively suitable. By introducing “Internet Plus” technologies, the informatization level of family doctor services can be rapidly enhanced. For example, leveraging big data platforms and artificial intelligence technologies can not only optimize health management processes but also compensate for the shortage of family doctor talent in the short term. Moreover, the application of information technology can significantly improve service efficiency and reduce resource wastage. The government can encourage the establishment of more patient self-education organizations through community outreach and policy support. These organizations can not only enhance patients’ awareness of health management but also alleviate the service burden on family doctors. The findings are relevant to other megacities, such as Guangzhou, Shenzhen, and Beijing. However, given the differences in resource allocation, policy environment, and community organization capabilities among these cities, the conclusions should be adapted to local contexts.

## Data Availability

The raw data supporting the conclusions of this article will be made available by the authors, without undue reservation.
