# Supplementary material for: Fuzzy-set qualitative comparative analysis of influencing factors on family doctor service performance during major public health emergencies
Source: Front Public Health. 2025 Apr 8;13:1565499. doi: 10.3389/fpubh.2025.1565499 (PMC12011841; doi:10.3389/fpubh.2025.1565499)
Supplement: Supplementary file 1 [file Table_1.DOCX]

**Summary of Conditions and Outcomes Considered in the Research Model**

**Table A1.** Summary of Conditions and Outcomes Considered in the Research Model

| **Condition/Outcome** | **Definition/Description** | **Inclusion Basis** | **Reference Sources** |
| --- | --- | --- | --- |
| **Outcome** |  |  |  |
| Performance of Family Doctor Services | Refers to the overall performance of family doctor teams in providing services, composed of effective contracting, effective services, and effective cost control. | Evaluating the performance of family doctor services during major public health emergencies can optimize resource allocation, enhance service effectiveness, and strengthen emergency medical response capabilities. | (Shanghai Municipal People’s Government, 2023)^1^ |
| **Technical Conditions** |  |  |  |
| Average Internet Medical Service Person-times | Refers to the total number of medical services conducted through internet platforms, including online consultations, remote diagnoses, online follow-ups, etc. | Reflects the coverage and usage frequency of family doctor services on the internet. | (Parker et al., 2021)  ^2^ |
| Information Technology Expenditure per Capita (/1000) | Refers to the total investment cost in information technology construction by medical institutions over a certain period, usually /1000, then divided by 1000 to obtain the specific value. | Reflects the investment intensity of medical institutions in information technology construction. | (Neves et al., 2021)^3^, (Rep. Courtney, 2020)^4^ |
| **Organizational Conditions** |  |  |  |
| Fiscal Allocation per Capita (/1000) | Refers to the average allocation income from government fiscal budgets per person, measured /1000. | Reflects the government's financial support for family doctor services. | (Higginson et al., 2020)^5^, (Australian Government Department of Health and Aged Care, 2020)^6^ |
| Family Doctor Team Members per Thousand Population | Refers to the total number of family doctor team members, usually counted and measured per thousand people. | Reflects the human resource allocation of family doctor services. | (British Medical Association, 2024) ^7^. |
| Medical Social Workers and Volunteers per Thousand Population | Refers to the total number of medical social workers and volunteers in community medical and health services, counted per thousand people. | Reflects the degree of community collaboration and resident participation, an important indicator for measuring community support networks. | (Moore, 2019; Schwarz et al., 2020)^8,9^, (Miao et al., 2021)^10^ |
| **Environmental Conditions** |  |  |  |
| Proportion of Elderly Population | Refers to the proportion of elderly people (usually defined as 60 or 65 years old and above) in the total population, used to measure the degree of population aging. | Very important for assessing the coverage and demand satisfaction of family doctor services among the elderly population. | (WHO, 2012)^11^, (A healthier future for all Australians : final report : June 2009 /, c2009.; Starfield et al., 2005)  ^12,13^ |
| Average Medical Expenses per Capita (/1000) | Refers to the average medical expense expenditure per person, usually measured /1000. | Reflects the economic burden and efficiency of family doctor services. | (Starfield et al., 2005; Starfield and Shi, 2002; Welfare;, 2018; WHO, 2008)^13–16^ |
| Number of Patient Self-Education Organizations per Thousand Population | Refers to the number of times patients are organized for self-education activities or the number of patients participating in a certain jurisdiction, counted per thousand people. | Reflects the efforts and effectiveness of family doctor services in improving patients' self-management abilities and health literacy. | (Huang et al., 2019)^17^ |

**References**

1. Shanghai Municipal People’s Government. Key Performance Assessment Indicators of Family Doctor Contracted Service in Shanghai (2022 Edition). January 19, 2023. Accessed January 3, 2025. https://www.shanghai.gov.cn/gwk/search/content/dadb49bc7ce5441fa22318ab3e21ea20

2. Parker RF, Figures EL, Paddison CA, Matheson JI, Blane DN, Ford JA. Inequalities in general practice remote consultations: a systematic review. *BJGP Open*. 2021;5(3):BJGPO.2021.0040. doi:10.3399/BJGPO.2021.0040

3. Neves AL, Li E, Gupta PP, Fontana G, Darzi A. Virtual primary care in high-income countries during the COVID-19 pandemic: Policy responses and lessons for the future. *Eur J Gen Pract*. 2021;27(1):241-247. doi:10.1080/13814788.2021.1965120

4. Rep. Courtney J [D C 2. H.R.748 - 116th Congress (2019-2020): CARES Act. March 27, 2020. Accessed January 2, 2025. https://www.congress.gov/bill/116th-congress/house-bill/748

5. Higginson S, Milovanovic K, Gillespie J, et al. COVID-19: The need for an Australian economic pandemic response plan. *Health Policy and Technology*. 2020;9(4):488-502. doi:https://doi.org/10.1016/j.hlpt.2020.08.017

6. Australian Government Department of Health and Aged Care. COVID-19 National Health Plan – Primary Health – Respiratory Clinics. March 12, 2020. Accessed January 2, 2025. https://www.health.gov.au/resources/publications/covid-19-national-health-plan-primary-health-respiratory-clinics?language=en

7. British Medical Association. Pressures in general practice data analysis. The British Medical Association is the trade union and professional body for doctors in the UK. November 29, 2024. Accessed January 4, 2025. https://www.bma.org.uk/advice-and-support/nhs-delivery-and-workforce/pressures/pressures-in-general-practice-data-analysis

8. Moore SM. Legitimacy, development and sustainability: understanding water policy and politics in contemporary China. *The China Quarterly*. 2019;237:153-173.

9. Schwarz G, Eva N, Newman A. Can public leadership increase public service motivation and job performance? *Public administration review*. 2020;80(4):543-554.

10. Miao Q, Schwarz S, Schwarz G. Responding to COVID-19: Community volunteerism and coproduction in China. *World Development*. 2021;137:105128. doi:https://doi.org/10.1016/j.worlddev.2020.105128

11. WHO. Global status report on noncommunicable diseases 2010. November 11, 2012. Accessed January 2, 2025. https://digitallibrary.un.org/record/706319?v=pdf

12. *A Healthier Future for All Australians : Final Report : June 2009 /*. National Health and Hospitals Reform Commission,; c2009. http://www.health.gov.au/internet/main/publishing.nsf/Content/83FD34219759A8EDCA2576000009A6C3/$File/Final_Report_of_the%20nhhrc_June_2009.pdf

13. Starfield B, Shi L, Macinko J. Contribution of primary care to health systems and health. *The milbank quarterly*. 2005;83(3):457-502.

14. Starfield B, Shi L. Policy relevant determinants of health: an international perspective. *Health Policy*. 2002;60(3):201-218. doi:10.1016/s0168-8510(01)00208-1

15. Welfare; AI of H and. *Australia’s Health 2018*. AIHW; 2018. https://www.aihw.gov.au/reports/australias-health/australias-health-2018

16. WHO. The World Health Report 2008 - Primary Health Care: Now More Than Ever. October 1, 2008. Accessed January 2, 2025. https://reliefweb.int/report/world/world-health-report-2008-primary-health-care-now-more-ever

17. Huang J, Zhang T, Wang L, et al. The effect of family doctor-contracted services on noncommunicable disease self-management in Shanghai, China. *Int J Health Plann Manage*. 2019;34(3):935-946. doi:10.1002/hpm.2865
