# Supplementary material for: Fuzzy-set qualitative comparative analysis of influencing factors on family doctor service performance during major public health emergencies
Source: Front Public Health. 2025 Apr 8;13:1565499. doi: 10.3389/fpubh.2025.1565499 (PMC12011841; doi:10.3389/fpubh.2025.1565499)
Supplement: Supplementary file 2 [file Table_2.DOCX]

**Measurement and Validation**

**Table B1.**Correlation Indicators for the Comprehensive Score of Family Doctor Performance in fsQCA

| **No.** | **Dimension** | **Indicator** | **Meaning** |
| --- | --- | --- | --- |
| 1 | Effective Signing | Contract Coverage Rate | Measures the extent of family doctor contract services among the target population. |
| 2 | Effective Services | Per Capita Standardized Electronic Health Record Count | Reflects the standardization of electronic health record establishment and management, indicating the level of informatization in family doctor services. |
|  |  | Cumulative Management Coverage Rate for Hypertension Patients | Measures the proportion of effectively managed hypertensive patients by year-end. |
|  |  | Cumulative Management Coverage Rate for Diabetic Patients | Measures the proportion of effectively managed diabetic patients by year-end. |
|  |  | Serious Mental Disorder Management Coverage Rate | Reflects the proportion of seriously mentally disordered patients managed effectively. |
|  |  | Tuberculosis Patient Health Management Coverage Rate | Measures the proportion of tuberculosis patients receiving health management. |
|  |  | Per Capita Total Beneficiary Count from Public Health Consultations | Proportion of the public benefiting from health consultations within a year. |
|  |  | Per Capita Family Health Service Visits | Proportion of family doctor services in overall health services. |
|  |  | Per Capita Fever Clinic Visits | Proportion of diagnostic and treatment services provided by fever clinics in total services. |
|  |  | Total Bed Days for Home Care Beds per Capita | Total bed days for home care patients within a year, calculated per thousand population. |
|  |  | Per Capita Population Served by Family Doctors | Proportion of the population covered by family doctor services. |
|  |  | Per Capita Total Beneficiary Count from Health Knowledge Lectures | Proportion of individuals benefiting from health knowledge lectures within a year. |
|  |  | Per Capita Total Count of Self-Education Organizations | Proportion of the population participating in self-education organizations. |
| 3 | Effective Cost Control | Per Capita Outpatient Visit Cost Reduction Count | Proportion of visits with reduced outpatient consultation fees in total visits. |
|  |  | Amount of Outpatient Visit Cost Reduction per Capita | Proportion of the total amount of reduced outpatient consultation fees. |
