# Supplementary material for: Fuzzy-set qualitative comparative analysis of influencing factors on family doctor service performance during major public health emergencies
Source: Front Public Health. 2025 Apr 8;13:1565499. doi: 10.3389/fpubh.2025.1565499 (PMC12011841; doi:10.3389/fpubh.2025.1565499)
Supplement: Supplementary file 3 [file Table_3.DOCX]

**Quality Control Measures for the Use of fsQCA Method**

**Table C1.** Quality Control Measures for the Use of fsQCA Method

| **No.** | **Content** | **Quality Control Method** |
| --- | --- | --- |
| 1 | Basis for Using fsQCA | 1. csQCA is suitable for binary variables, fsQCA for continuous variables, and mvQCA for multi-categorical variables. This study uses fsQCA due to the continuous nature of the variables.2. fsQCA is appropriate for medium-sized samples (34 cases in this study).3. fsQCA extends beyond csQCA's limitation of binary variables, entering the realm of interval variables, and can explain nonlinear and complementary associations. |
| 2 | Theoretical Foundation of Conditions and Outcomes | The conditions and outcomes anchored in the TOE model provide a solid theoretical foundation for this study. |
| 3 | Complementarity with Other Methods | This study complements qualitative research on "the dilemma and influencing factors of family doctor service provision based on street-level bureaucracy theory," offering empirical analysis-based strategies and policy recommendations for future work. |
| 4 | Relevant Cases | 1. Prior to this study, extensive domestic and international research was conducted on "the service provision pathways of family doctors during major public health emergencies," with topic clustering via latent Dirichlet allocation.2. This study explores the differences in family doctor service performance during emergencies, explaining how different combinations of conditions can lead to the same outcomes. |
| 5 | Number of Conditions | Maintaining the ratio of conditions to cases within the range recommended by guidelines (34 cases and 8 conditions) ensures the reliability of fsQCA. |
