# Supplementary material for: Fuzzy-set qualitative comparative analysis of influencing factors on family doctor service performance during major public health emergencies
Source: Front Public Health. 2025 Apr 8;13:1565499. doi: 10.3389/fpubh.2025.1565499 (PMC12011841; doi:10.3389/fpubh.2025.1565499)
Supplement: Supplementary file 4 [file Table_4.DOCX]

**Necessity Analysis**

**Table D1.** Analysis of Necessary Conditions for Overall Community Family Doctor Service Performance

| **Condition Variable** | **High-Level Family Doctor Service Performance** | | **Non-High-Level Family Doctor Service Performance** | |
| --- | --- | --- | --- | --- |
|  | **Consistency** | **Coverage** | **Consistency** | **Coverage** |
| High Average Internet Medical Service Person-times | 0.518 | 0.641 | 0.533 | 0.638 |
| Non-High Average Internet Medical Service Person-times | 0.707 | 0.610 | 0.700 | 0.584 |
| High Information Technology Expenditure per Thousand Population (/1000) | 0.709 | 0.744 | 0.526 | 0.534 |
| Non-High Information Technology Expenditure per Thousand Population (/1000) | 0.556 | 0.548 | 0.748 | 0.714 |
| High Fiscal Allocation per Capita (/1000) | 0.754 | 0.766 | 0.552 | 0.543 |
| Non-High Fiscal Allocation per Capita (/1000) | 0.550 | 0.559 | 0.763 | 0.750 |
| High Family Doctor Team Members per Thousand Population | 0.745 | 0.768 | 0.606 | 0.604 |
| Non-High Family Doctor Team Members per Thousand Population | 0.616 | 0.618 | 0.768 | 0.745 |
| High Medical Social Workers and Volunteers per Thousand Population | 0.726 | 0.802 | 0.496 | 0.530 |
| Non-High Medical Social Workers and Volunteers per Thousand Population | 0.575 | 0.541 | 0.815 | 0.742 |
| High Proportion of Elderly Population | 0.836 | 0.879 | 0.433 | 0.440 |
| Non-High Proportion of Elderly Population | 0.468 | 0.460 | 0.881 | 0.839 |
| High Average Medical Expenses per Capita (/1000) | 0.848 | 0.894 | 0.434 | 0.443 |
| Non-High Average Medical Expenses per Capita (/1000) | 0.471 | 0.463 | 0.896 | 0.851 |
| High Number of Patient Self-Education Organizations per Thousand Population | 0.690 | 0.727 | 0.535 | 0.545 |
| Non-High Number of Patient Self-Education Organizations per Thousand Population | 0.568 | 0.558 | 0.733 | 0.696 |

**Table D2.** Analysis of Necessary Conditions for Family Doctor Service Performance in Central Urban Communities

| **Condition Variable** | **High-Level Family Doctor Service Performance** | | **Non-High-Level Family Doctor Service Performance** | |
| --- | --- | --- | --- | --- |
|  | **Consistency** | **Coverage** | **Consistency** | **Coverage** |
| High Average Internet Medical Service Person-times | 0.586 | 0.810 | 0.650 | 0.463 |
| Non-High Average Internet Medical Service Person-times | 0.612 | 0.773 | 0.734 | 0.477 |
| High Information Technology Expenditure per Thousand Population (/1000) | 0.686 | 0.805 | 0.775 | 0.468 |
| Non-High Information Technology Expenditure per Thousand Population (/1000) | 0.547 | 0.825 | 0.678 | 0.527 |
| High Fiscal Allocation per Capita (/1000) | 0.690 | 0.878 | 0.712 | 0.467 |
| Non-High Fiscal Allocation per Capita (/1000) | 0.581 | 0.797 | 0.814 | 0.575 |
| High Family Doctor Team Members per Thousand Population | 0.646 | 0.977 | 0.724 | 0.564 |
| Non-High Family Doctor Team Members per Thousand Population | 0.711 | 0.833 | 0.971 | 0.586 |
| High Medical Social Workers and Volunteers per Thousand Population | 0.674 | 0.889 | 0.650 | 0.442 |
| Non-High Medical Social Workers and Volunteers per Thousand Population | 0.577 | 0.762 | 0.837 | 0.570 |
| High Proportion of Elderly Population | 0.874 | 0.898 | 0.761 | 0.403 |
| Non-High Proportion of Elderly Population | 0.419 | 0.773 | 0.807 | 0.767 |
| High Average Medical Expenses per Capita (/1000) | 0.838 | 0.888 | 0.729 | 0.398 |
| Non-High Average Medical Expenses per Capita (/1000) | 0.433 | 0.756 | 0.796 | 0.716 |
| High Number of Patient Self-Education Organizations per Thousand Population | 0.726 | 0.819 | 0.734 | 0.427 |
| Non-High Number of Patient Self-Education Organizations per Thousand Population | 0.492 | 0.782 | 0.690 | 0.564 |

**Table D3.** Analysis of Necessary Conditions for Family Doctor Service Performance in Non-Central Urban Communities

| **Condition Variable** | **High-Level Family Doctor Service Performance** | | **Non-High-Level Family Doctor Service Performance** | |
| --- | --- | --- | --- | --- |
|  | **Consistency** | **Coverage** | **Consistency** | **Coverage** |
| High Average Internet Medical Service Person-times | 0.369 | 0.370 | 0.465 | 0.916 |
| Non-High Average Internet Medical Service Person-times | 0.917 | 0.466 | 0.680 | 0.679 |
| High Information Technology Expenditure per Thousand Population (/1000) | 0.761 | 0.647 | 0.382 | 0.638 |
| Non-High Information Technology Expenditure per Thousand Population (/1000) | 0.574 | 0.321 | 0.789 | 0.866 |
| High Fiscal Allocation per Capita (/1000) | 0.894 | 0.631 | 0.459 | 0.636 |
| Non-High Fiscal Allocation per Capita (/1000) | 0.483 | 0.313 | 0.733 | 0.932 |
| High Family Doctor Team Members per Thousand Population | 0.963 | 0.584 | 0.538 | 0.640 |
| Non-High Family Doctor Team Members per Thousand Population | 0.407 | 0.310 | 0.651 | 0.972 |
| High Medical Social Workers and Volunteers per Thousand Population | 0.839 | 0.683 | 0.407 | 0.650 |
| Non-High Medical Social Workers and Volunteers per Thousand Population | 0.570 | 0.329 | 0.802 | 0.907 |
| High Proportion of Elderly Population | 0.754 | 0.834 | 0.243 | 0.529 |
| Non-High Proportion of Elderly Population | 0.574 | 0.279 | 0.924 | 0.880 |
| High Average Medical Expenses per Capita (/1000) | 0.872 | 0.906 | 0.264 | 0.538 |
| Non-High Average Medical Expenses per Capita (/1000) | 0.556 | 0.278 | 0.954 | 0.936 |
| High Number of Patient Self-Education Organizations per Thousand Population | 0.613 | 0.563 | 0.420 | 0.757 |
| Non-High Number of Patient Self-Education Organizations per Thousand Population | 0.735 | 0.392 | 0.758 | 0.793 |
